# Orchestrating Multi-Agent Knowledge Ecosystems: The Role of Makerspaces

**DOI:** 10.3389/fpsyg.2022.898134

**Published:** 2022-05-18

**Authors:** Jia-Lu Shi, Guo-Hong Chen

**Affiliations:** Management College, Fujian University of Technology, Fuzhou, China

**Keywords:** multi-agent, innovation, knowledge ecosystem, makerspace, orchestrating process

## Abstract

In the knowledge economy, the process of knowledge sharing and creation for value co-creation frequently emerge in a multi-agent and multi-level system. It's important to consider the roles, functions, and possible interactive knowledge-based activities of key actors for ecological development. Makerspace as an initial stage of incubated platform plays the central and crucial roles of resource orchestrators and platform supporter. Less literature analyses the knowledge ecosystem embedded by makerspaces and considers the interactive process of civil society and natural environment. This study constructs a multi-agent and multi-level knowledge ecosystem from macro, meso, and micro perspective based on Quintuple Helix theory and designs four evolutionary stages of knowledge orchestrating processes. This study finds that the symbiosis, co-evolution, interaction, and orchestration of multiple agents in the knowledge ecosystem should be merged with each other for value co-creation, which helps to take a systematic approach for policymakers, managers, and researchers.

## Introduction

**Figure 1:**
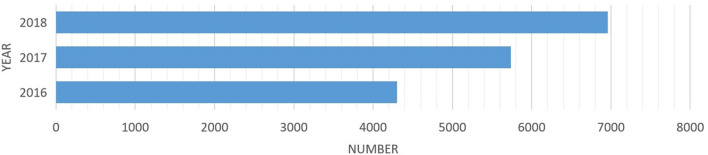
The number of makerspaces in 2016−2018.

**Figure 2:**
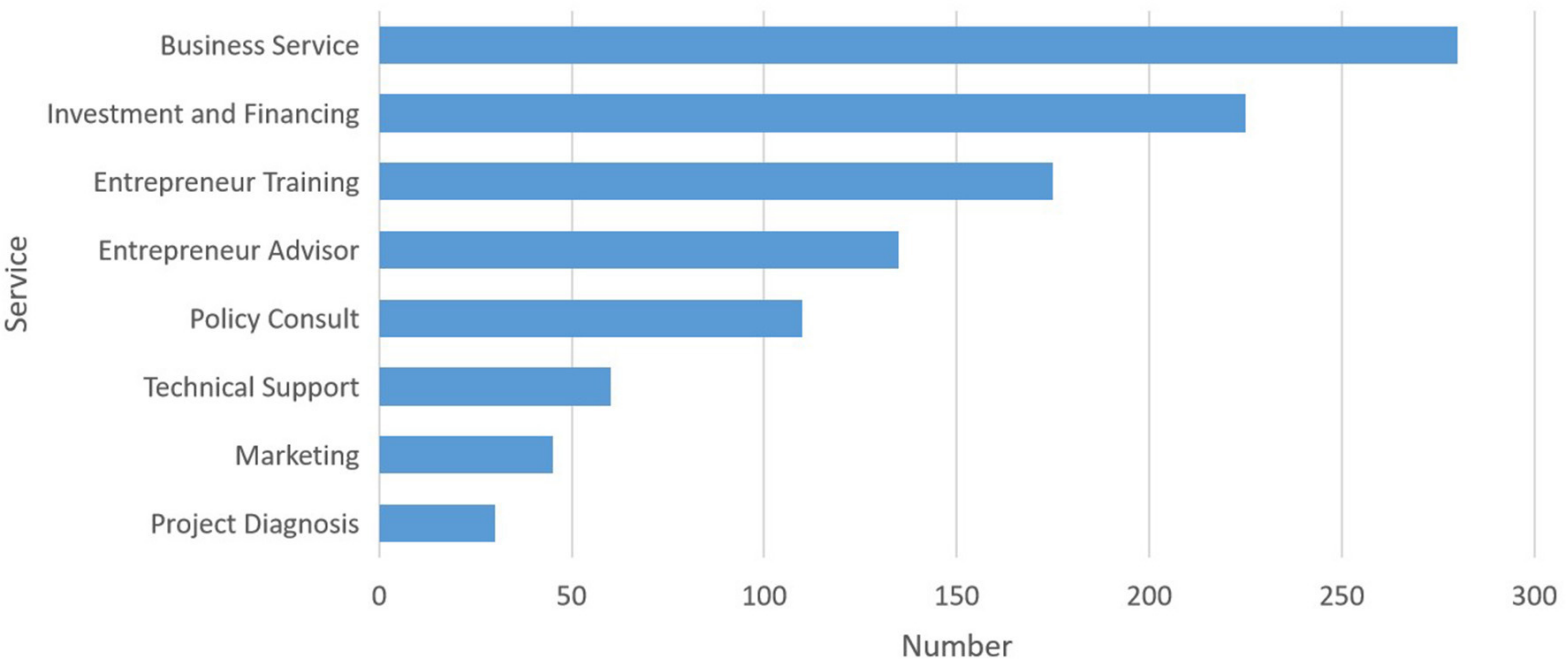
The services provided by makerspaces.

**Figure 3:**
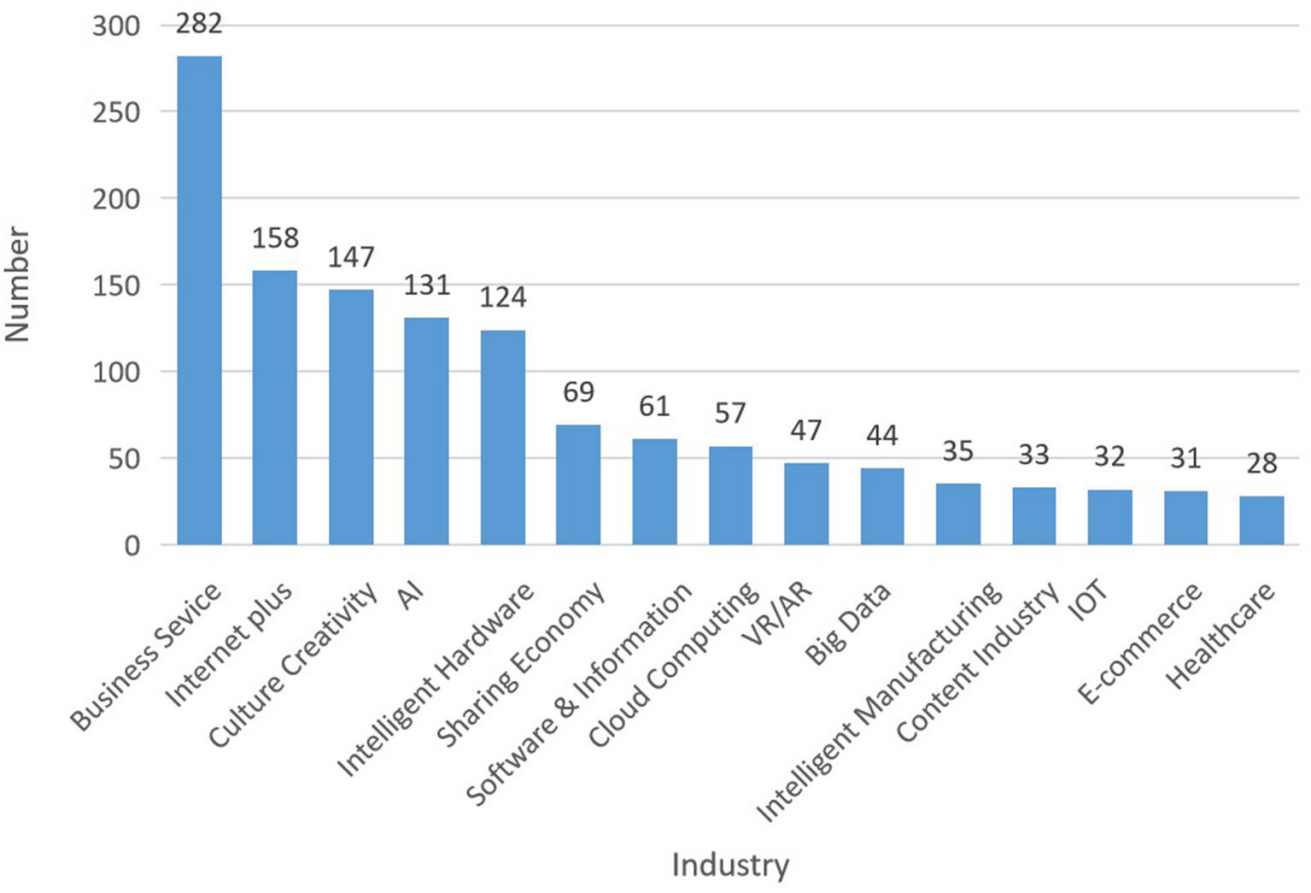
The distribution of associated industries with makerspaces. Plus: indicates that the internet is utilized in the various industries.

**Figure 4:**
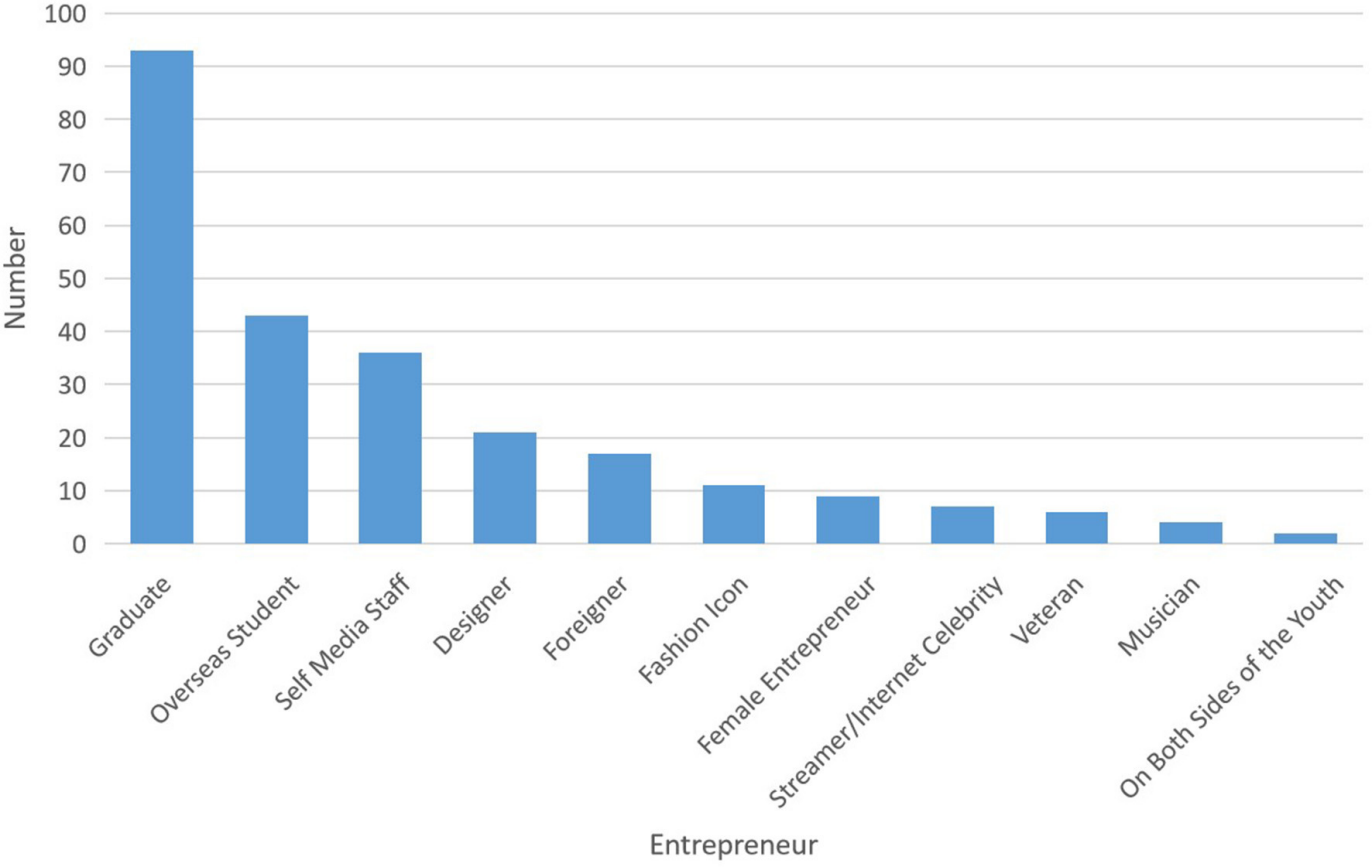
The distribution of entrepreneurs facilitated by makerspaces.

The knowledge ecosystem is a non-linear and complex system that focuses on the knowledge interaction between major systems and subsystems (Carayannis and Campbell, 2009). The process of knowledge sharing and creation for value co-creation frequently emerge in a comprehensive system that includes pluralized and advanced knowledge shared among organizations and agents in the co-evolution and mutual learning spectrum (Adner and Kapoor, 2010). It's important to consider the possible interactive knowledge-based activities of key stakeholders for ecological development. In response to a national strategy of “mass entrepreneurship and innovation for all,” makerspaces in China flourished in recent years and developed rapidly. Presently, the government has invested an abundance of funds to support the development of makerspaces. According to the data released by the Torch High Technology Industry Development Center, Ministry of Science and Technology, there were 6,959 makerspaces in China (including in the Torch program) by the end of 2018, ranking first in the world 2 years in a row (Figure 1). The services provided by makerspaces are increasingly seen in professional areas, such as business services, investment and financing, entrepreneur training, entrepreneur adviser, policy consultant, technical support, marketing, project diagnosis, and so on (Figure 2). On October 15, 2018, “White Paper of Makerspaces in China” was published, which showed that deeper penetration and integration of makerspaces are associated with multiple industries such as business services, internet+, cultural creativity, AI, intelligent hardware, sharing economy, software and information, cloud computing, VR/AR, big data, intelligent manufacturing, content industry, IOT, e-commerce, and healthcare, etc. (Figure 3). In addition, makerspaces facilitate entrepreneurs such as graduates, overseas students, self-media staff, designer, foreigner, fashion icon, female entrepreneur, streamer, internet celebrity, veteran, musician, on both sides of youth, etc. (Figure 4).

Makerspaces are the product of knowledge economy that pulls from a variety of industries and are also pushed by other key organizations, crowds, and industries. A knowledge ecosystem encourages symbiosis, resource orchestration, and value co-creation, which transform persons from knowledge users to knowledge sharers and then knowledge creators (Lindtner, 2014). Makerspaces are inevitably embedded into the ecosystem as an element, a community or a population as a crucial role (Légaré et al., 2014). Previous research demonstrates that the internal interaction of makers and startup teams is critical in creating value. However, the literature largely overlooked that the orchestrating process of a knowledge ecosystem also depends on its external alignment, determined by the broader political, economic, educational, societal, and natural environments. In the knowledge ecosystem, collaborative symbiosis has become a new mode of the relationship between governments, enterprises, universities, citizens, and the natural environment, forming knowledge-based corporate activities. The orchestrating process of knowledge ecosystem is the set of deliberate and purposeful actions constructed by multiple actors as they seek to share, integrate, diffuse, and create knowledge and value (Dhanaraj and Parkhe, 2006). In this context, makerspaces are bound to become hub agents to embed in knowledge ecosystem (Yang et al., 2019). Different from the original incubators, accelerators, high-tech parks, and traditional industrial clusters, the definition of makerspace implies that it is open, convenient, and low-cost through specialized entrepreneurial services where the knowledge exchange and sharing is built in among the interactive system. To create a project, it's necessary to share ideas, to analyze the market, and manage the startup team in internal makerspaces, and also necessary to exchange information with various external stakeholders.

This paper stands to make significant contributions. Firstly, this study establishes a theoretical framework for a multi-level and multi-agent knowledge ecosystem embedded by makerspaces-centric from macro, meso, and micro perspectives. Secondly, this research elaborates the roles, functions, and interactive activities of governments, universities, businesses, civil society, and natural environment with makerspaces as the roles of resource orchestrator and platform supporter based on Quintuple Helix theory. Thirdly, this study suggests four different evolutionary stages of knowledge exchange, coordination, cooperation, and orchestration. Finally, this research adopts a case study research to verify the processes of knowledge orchestration and recommends some implications for policymakers, managers, and researchers.

## Theoretical Background and Literature Review

### Makerspaces Embedded in Knowledge Ecosystem

Makerspaces are physical spaces located on campuses, libraries, museums, and other community settings where makers can communicate, exchange, create, and solve problems through the incubated platform (Secundo et al., 2020). Makers usually pay fees to utilize the space, services, and sharing tools (e.g., 3D printers, metalworking equipment, woodworkers, etc.; Shah et al., 2019). They participate in knowledge sharing activities to meet and collaborate with diverse industries and enterprises. Makerspace is the headstream of a chained incubated platform. A considerable part of research on makerspaces development has occurred outside of the academic sphere. Therefore, previous researches defined three classifications of incubated platforms abroad (Makerspace, Hackerspace, Fab lab; Table 1) and in China (Makerspace, Incubation, Accelerator) (Pauwels et al., 2016; Table 2). Hence this paper designs the research scope of makerspace as the primary incubation in the pre-startup phase excluding the incubation and the accelerator.

**Table 1:** Three classifications of incubated platforms abroad.

**Classification**	**User**	**Function**	**Activity**
Makerspace	Maker	Access to open spaces, tools, and equipment.	Design, prototyping, sharing, communication, creation for makers.
Hackerspace	Hacker	Access to digital technology, electronic art, and other technologies.	Sharing, meeting, working and cross-learning for community-operated space.
Fab lab	Civil	Access to technical prototyping.	Innovation, invention, and stimulus for local entrepreneurship.

*Source: A summary based on (Pauwels et al., 2016)*.

**Table 2:** Three classifications of incubated platforms in China.

**Classification**	**Definition**	**User**	**Function**
Makerspace	Primary incubation located on campuses, libraries, museums, and some other community settings where makers can communicate, exchange, create, and solve problems for makers.	Maker emphasis on testing unproven ideas and identifying product-market fit in the pre-startup phase.	Foster the making of projects by providing specific services such as advisory consultation, financial investment, and knowledge sharing in addition to sharing tools.
Incubation	Advanced incubation as a typically and traditionally physical space, available on relatively flexible terms that provide additional incubation services.	New and small businesses through the early stages of development in the advanced-incubation phase.	Provide services including mentorship, entrepreneurial training, technical facilities such as laboratory equipment, and selective admission (but typically less so than accelerators).
Accelerator	Mature incubating programs that sometines do not offer physical space but aim to provide further services remotely.	More established and high growth-driven companies looking to scale up their business in the pre-accelerator phase.	Provide services including assistance in developing the business plan, investor pitch deck, prototypes, and initial market testing through a highly selective and cohort-based program.

*Source: A summary based on (Pauwels et al., 2016)*.

Based on the study of the ecological system theory (Moore, 1993), the ecosystem between enterprises is a kind of “economic community based on organizational interaction,” and it is a dynamic structural system composed of organizations or groups for the purpose of value co-creation (Yun and Liu, 2019). The existing research of ecosystem can be clarified as comprising four main research perspectives. The first is the industrial ecology perspective, which is focused on the industrial ecosystem (Tsujimoto et al., 2018; Hofmann and Giones, 2019; Parida et al., 2019; Fraccascia et al., 2021). The second is the business ecosystem perspective (Ma et al., 2018; Rinkinen and Harmaakorpi, 2018; Senyo et al., 2019; Hult et al., 2020), from which some renowned scholars focus on platform management (Sun et al., 2020). The third is the knowledge ecosystem based on social network theory and resource-based view theory (Miller et al., 2017; Järvi et al., 2018; Entezari, 2020). The multi-agent knowledge ecosystem is increasingly significant in the field of knowledge management. The agents interplay with each other by various means of sharing resources, including tacit or explicit knowledge, information, contracts, trust, benefits, and goals. Unlike traditional industrial and business systems that pursue profit maximization, this differs in the ecological characteristics that orchestrate continuous balance between human and non-human organics (Stilgoe et al., 2013). A knowledge ecosystem aims to mainly drive co-value for individuals, sectors, and nations (Ritala and Almpanopoulou, 2017).

### Orchestrating Process of Multiple Agents

It is difficult to complete an innovation activity independently, given that frontier information, knowledge, and technology are distributed among a complex system of multiple agents. Therefore, makerspaces should achieve and utilize a variety of knowledge from multiple agents, including state or government, university, industry or business, and civil or public to help launch new entrepreneurial opportunities (Carayannis et al., 2018a). In the knowledge ecosystem makerspaces play the central role, which could be called knowledge intermediaries and knowledge gatekeepers that not only share information but also build knowledge linkage (Sieg et al., 2010). The makerspace is generally defined as an organization that provides a supportive role for collaboration during evolutionary stages from existing knowledge collection to new knowledge creation (Howells, 2006). The functions extend from collaborating with agents to bridging a wide linkage of information, knowledge and technology in a further process of co-creation and co-development (Smedlund, 2006; Klerkx and Leeuwis, 2008a,b; Boon et al., 2011; Edler and Yeow, 2016). Managers of makerspaces may work on profit sharing collaboratively and they are generally willing to help makers and startups to incubate products and services. It is noteworthy that makerspaces have a degree of nonprofit characteristics of social enterprises in China, which mainly inspire social responsibility to create greater value for society and natural ecology. The emergence of makerspaces enhances innovative awareness and stimulates enthusiasm for mass entrepreneurship. Also, makerspaces increase the employment rate and provide more jobs to graduates. Thus, makerspaces have tight connection with civil society and environment, which could facilitate financial and non-financial interaction to their users (Huyghe et al., 2014).

In the knowledge sharing society, it's difficult to solve with the solutions independently. Therefore, the knowledge ecosystem comprises multiple agents and emerges as knowledge orchestration for value co-creation. A knowledge ecosystem, as an emerging environment for achieving orchestration process, has been widely recognized (Silvestre and Tîrcă, 2019). The knowledge ecosystem is like a tropical rainforest in which multiple agents evolve, cooperate, and compete (Boons et al., 2013; Klewitz and Hansen, 2014; Oksanen and Hautamäki, 2015). Presently, the in-depth studies of multi-agent orchestration process are insufficient. Most scholars regard the knowledge orchestrating process as an open and inclusive system. For example, Carayannis and Campbell (2009) believe that the knowledge orchestration includes a wider network environment; Schiuma and Lerro (2010) define the knowledge orchestration as resource sharing system for the purpose of creating joint value. There is lack of academic research on the evolutionary process of knowledge orchestration in the ecosystem which could help scholars stimulate and predict the developing trends of makerspaces.

### Quintuple Helix

**Figure 5:**
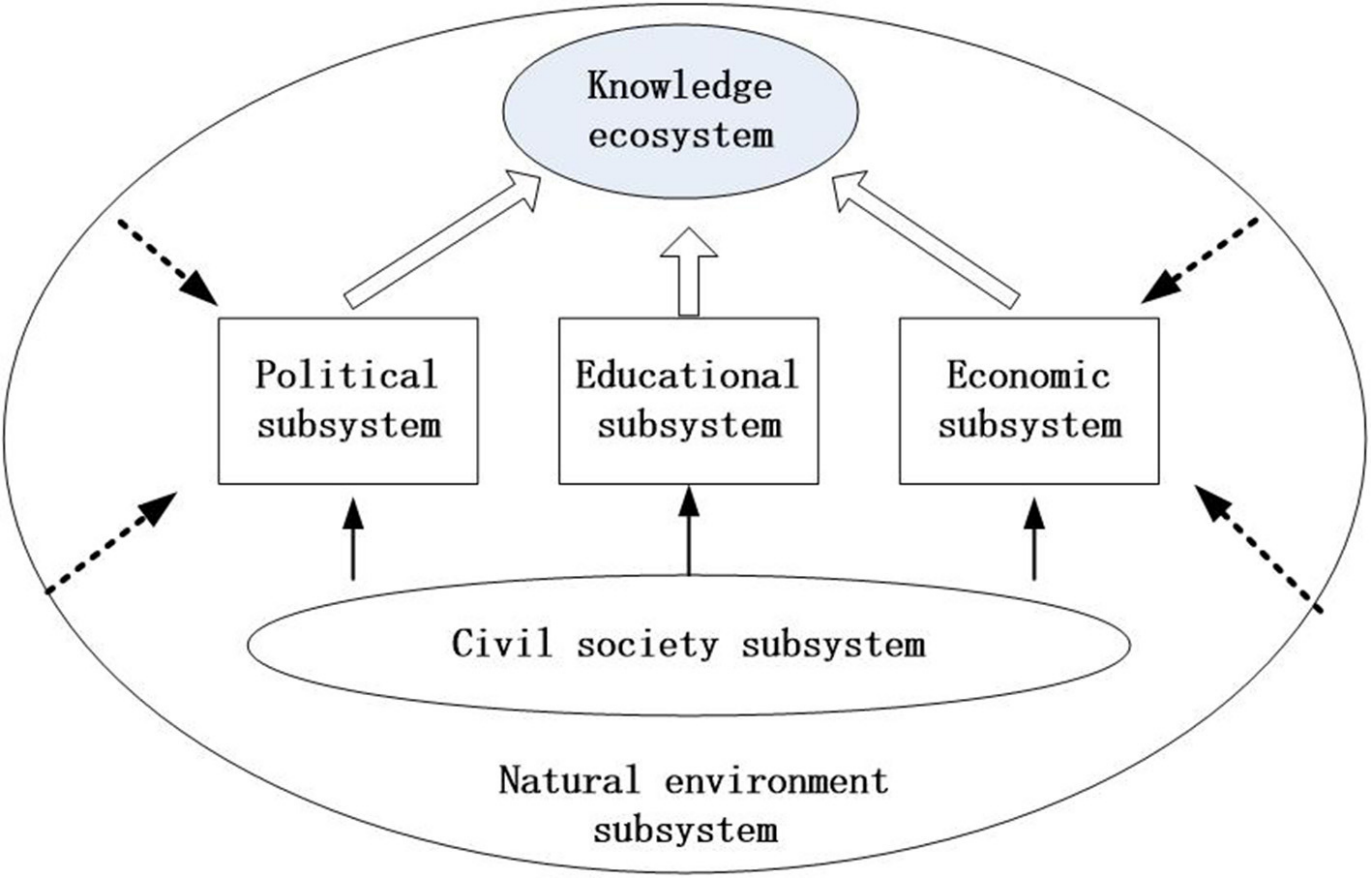
The relationship of knowledge ecosystem and subsystems.

Quintuple helix theory evolved from Triple Helix theory, Quadruple Helix theory and further to N-Helix theory, demonstrating helical interaction forms among multiple agents. Triple Helix theory, initiated by Etzkowitz and Leydesdorff (2000), describes a non-linear innovation model including governments, universities, and industries. It is the traditional and common model for the innovative interaction with multiple agents for researchers. For Etzkowitz and Leydesdorff (2000), the trilateral and hybrid organizations of university-industry-government relations are crucial, with universities representing the core role in the knowledge society. Quadruple Helix theory, connecting top-down policies and regulations with bottom-up grassroots initiatives, expands the role of civil society, media, and the culture-based public to the Triple Helix model (Carayannis and Campbell, 2009). In the natural sciences, an ecosystem is defined as a set of one or more communities of living organisms and non-living organisms interacting with each other (Odum and Barrett, 1971). With the global warming environment and growing natural resource depletion, effects on the natural environment should be considered. The Quintuple Helix system adds a fifth helix in the wider context of non-human agents, the “natural environment or natural environments of society and economy,” which implies the issues of sustainable development and orchestrating process. In the multi-level framework of the Quintuple Helix model that relates to the knowledge ecosystem, knowledge orchestrator and natural environments are introduced. In this study the quintuple helix ecosystem is designed to facilitate innovation, knowledge, and resource orchestration that is composed of five subsystems (educational subsystem, economic subsystem, political subsystem, civil society, and natural environment) relating to five helices (Carayannis et al., 2012; Table 3). Each actor in the sub-ecosystem provides special resources to the ecosystem, but also integrates, transforms, and utilizes resources to solve interactive challenges to each other. The relationship of each subsystem in the Quintuple Helix system is presented in Figure 5. Political subsystem, educational subsystem, and economic subsystem are the three classical systems to support the knowledge ecosystem. Civil society subsystem as the role of mass participants connects with knowledge ecosystem mainly through the linkages to political, educational, and economic subsystems. Natural environment subsystem as a non-living agent usually provides the unpredictable evens and natural resources to knowledge ecosystem and other subsystems.

**Table 3:** Main interpretations of five helices.

**Name**	**Agent**	**Subsystem**	**Interpretation**
First helix	State/ Government	Political subsystem	Provide political and legal support (e.g., laws, clearances, policy, and public goods).
Second helix	Academia/ University	Educational subsystem	Generate and disseminate new knowledge (e.g., new know-how of an agent).
Third helix	Industry/ Business	Economic subsystem	Control, possess, and generate economic capital (e.g., machines, money, etc.).
Fourth helix	Media-based and culture-based public/ Civil society/ Arts, artistic research and arts-based innovation	Civil society subsystem	Dominate social capital (e.g., solidarity, lifestyle, friendships, etc.) through culture (e.g., tradition, values, etc.) and media-based public (e.g., news).
Fifth helix	Natural environment/ Natural environments of society and economy	Natural environment subsystem	Conserve natural capital (e.g., natural resources, climate, air quality, geological stability) / the socio-ecological reliably environment based on knowledge production (research) and knowledge application (innovation) that consider environmental issues.

Generally, there is no consensus on the academic research of makerspaces embedded in the knowledge ecosystem based on Quintuple helix theory. A large proportion of literature addresses the interactive activities of knowledge exchange, sharing, dissemination, creation, and application among the multiple agents, and makerspaces have strong knowledge-based linkages to governments, universities, and businesses (Klerkx and Leeuwis, 2008c; Lichtenthaler and Lichtenthaler, 2009). Nonetheless, with the deterioration of the natural environment, the deficient resources, and the advanced capability of cooperative innovation, makerspaces are the inevitable product of the continuous evolutionary process in the knowledge ecosystem associated with other agents such as citizens, public, media, and natural environment that coincide with a Quintuple Helix system. Yet little research elaborates systematically how makerspaces create and extract effective knowledge for value co-creation, that is, how multiple agents orchestrate with each other. We believe a specific and explicit framework or conceptual model is essential for the mutual reinforcement and reciprocal relationships. That is the focus of this study.

## Developing Multi-Agent Orchestrating Process of Knowledge Ecosystem

It is a process of exploration and explanation to study knowledge ecosystem embedded by makerspaces-centric from the quintuple helix perspective. Therefore, this paper adopts the research methods of in-depth semi-structured expert interviews and case study, which help the researchers to find important information not covered in the literature review (Kallio et al., 2016). Thirty experts were interviewed constructed by chief manager in makerspaces, public officials in managing sectors, chairmen of related associations and alliance, leaders of startup teams with the support of MOST (Ministry of Science and Technology) in China. The period of the survey was from April 12th, 2020 to July 20th, 2020. The description of interviewed experts is represented in Table 4.

**Table 4:** Description of experts.

**Index**	**Category**	**Number of**	**Percentage (%)**
		**respondents**
Gender	Male	22	73.33
	Female	8	26.67
Age	20–30	5	16.67
	30–40	20	66.67
	40–50	3	10.00
	50–60	2	6.66
Years of working	5–10	5	16.67
	10–15	22	73.33
	16–20	3	10.00
Organizational positions	Chief manager of makerspaces	15	50.00
	Public officials	3	10.00
	Chairmen of associations	2	6.67
	Leaders of startup teams	10	33.33

**Figure 6:**
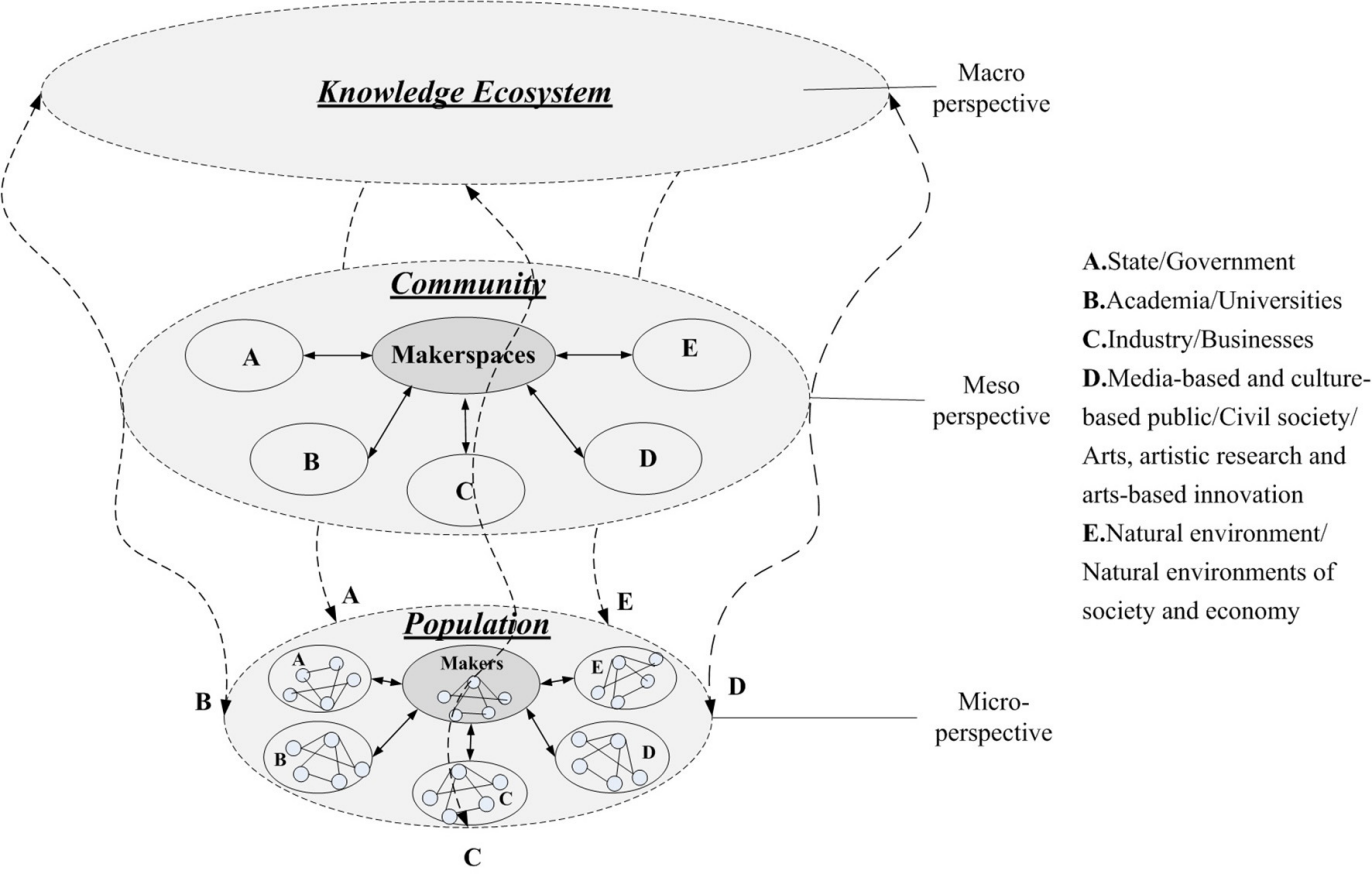
Multi-level and multi-agent model of knowledge ecosystem (lateral view).

Based on experts' suggestions, this section explores the roles, functions, interactive activities of makerspaces as a central role in knowledge ecosystem from macro, meso, and micro perspectives and develops a framework of knowledge orchestrating process based on Quintuple Helix theory. The original model is shown in Figure 6. This study explores three main attributes from ecological perspective: multi-level ecological balance, multi-agent helical interaction, and multi-stage orchestrating process.

### Multi-Level Ecological Balance

Biological metaphor is the basic hypothesis of ecological systems, which discerns the social and economic agents and milieu from a biological perspective (Farooq, 2019). It identifies the producers, consumers, and decomposers, and discusses the interaction and balance among communities, populations and the natural environment. Similarly, the knowledge also comprises the knowledge ecosystem, knowledge community, and knowledge population. The knowledge universe has evolved enormously with increasing R&D investment, technological globalization, information explosion, and internet popularization. The evolutionary ecology of knowledge, due to the simultaneous emergence of various knowledge sharers, drivers, disseminators, communicators, and enablers based on sharing economy, is inducing mass innovation and entrepreneurship. The emergence of makerspaces is an important mutual supplement in the knowledge ecosystem and the orchestrator of innovative resources in the knowledge universe. Table 5 represents the comparison of knowledge ecosystem with natural ecosystem. From the macro, meso, and micro perspectives, there are three levels of knowledge ecosystem: ecosystem, community, and population. Each component of the ecosystem often plays the dual roles of the agent and the environment simultaneously at different levels. As Figure 6 depicts, the five circles of A, B, C, D, and E demonstrate the five key agents at the meso and micro levels. Also, the five spiral lines of A, B, C, D, and E indicate the key agents that interact with each other across the macro, meso, and micro levels based on a Quintuple Helix model.

**Table 5:** Comparison of knowledge ecosystem with natural ecosystem.

**Natural ecosystem**	**Definition**	**Element**	**Knowledge ecosystem**	**Definition**	**Element**
Ecosystem	The biotic community and its living environment as an interacting system.	• Evolution, • Energetics, • Adaptation, • Mutation, • Food webs, Biogeochemical flow, • Predation, • Consumers, • Efficiency mutualism, Landscape	Knowledge ecosystem	A complex adaptive system.	• Knowledge cycle, • Informatics, • Managing change, • Innovation, • Knowledge networks, Communication flow, Acquisition, • Knowledge users, • Strategic alliances, Organizational culture
Community	A set of populations dominating an ecosystem.		Organization	A bundle of resources and competencies.	
Population	Members of an ecological community.		Competency	Knowledge artifacts involving cognitive, tacit and explicit elements.	

The macro level of knowledge ecosystem includes non-biological milieu, producers, consumers and decomposers (Carayannis et al., 2018b). Makerspaces play a central role in the ecosystem at the macro-level, which orchestrate the knowledge subjects and elements in a wider range across different levels. The multi-level model realizes the cross-layer flow and sharing of elements through the makerspaces as the intermediary and orchestrator (Bashir and Farooq, 2019). From the perspective of meso knowledge ecosystems, makerspaces play the role of community, which means the assemblage and mixture of diverse populations in a certain region. As a incubated platform, makespaces assemble makers, startups, and entrepreneurs. At the micro-level of knowledge ecosystems, makerspaces provide the economic and social environment for knowledge-based interactive activities, such as capital, technology, advisor, and culture, and also construct an intermediary bridge to educational institutions, scientific research institutes, government and public sectors, investment and financing institutions, intermediary organizations, vertical industrial chain enterprises, and civil society. The multi-level knowledge ecosystem is a complicated system along with multi-agents spiraling up. As an integration of knowledge elements, agents and the environment, makerspaces play the roles of resource orchestrator and platform supporter to drive the knowledge sharing and orchestration for long-term ecological balance. According to a biological theory, the essential functions are substance circulation, energy exchanges, and information transfer in the process of ecological balance. This study mainly focuses on the external interactive and collaborative relationships among multiple agents at the meso level and analyzes the activities of knowledge dissemination, exchange, sharing, and creating at the same level and across the levels in the knowledge ecosystem framework.

### Multi-Agent Helical Interaction

**Figure 7:**
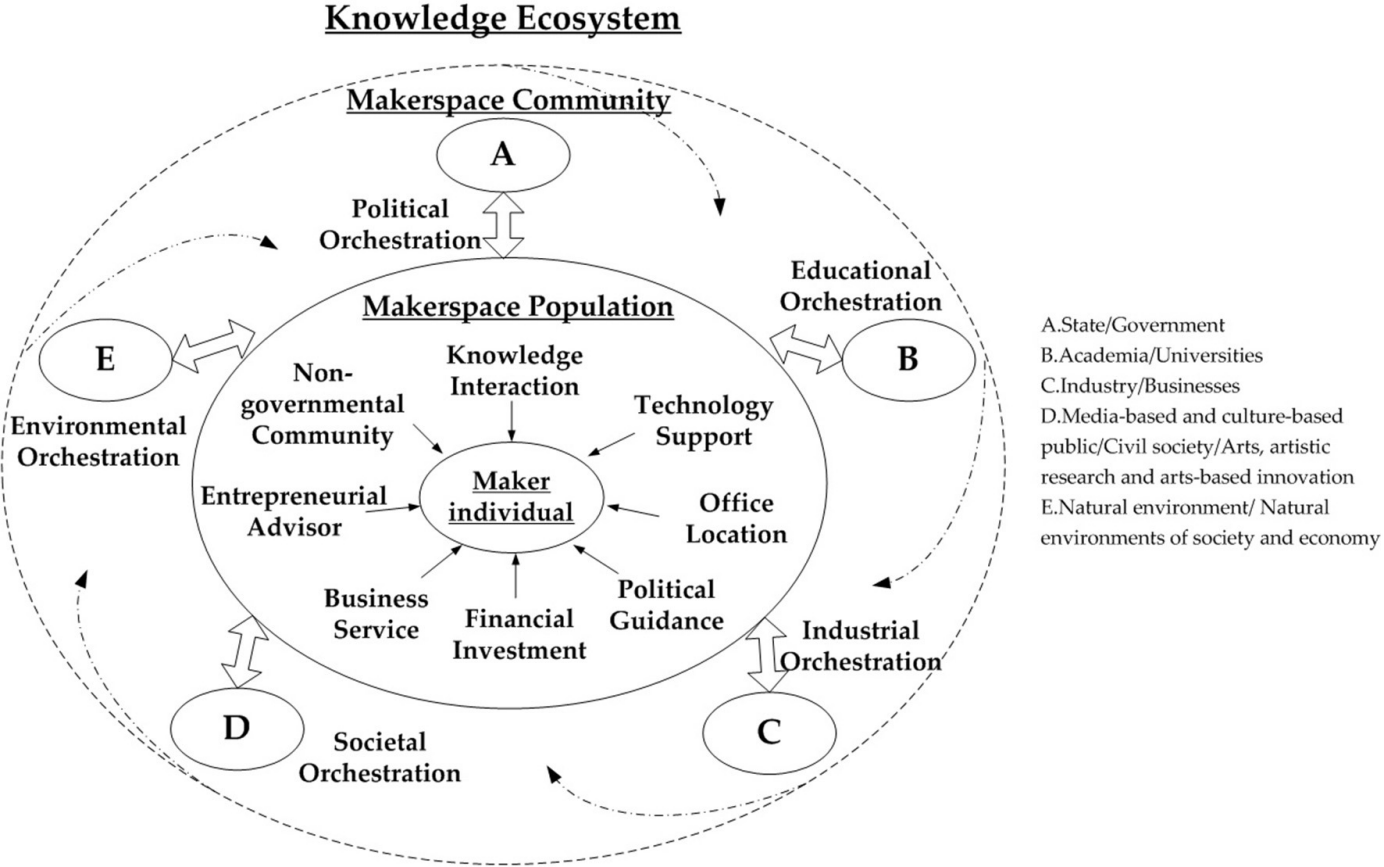
Multi-level framework of knowledge ecosystem (top view).

Interactive activities in the knowledge ecosystem involve various agents co-creating knowledge together across different levels of ecosystem, community, and population (Figure 7). The environment should be regarded as a knowledge atmosphere, information, and resource dissemination. The key agents of industries, governments, universities, civil society, and natural environment demonstrate interaction and co-evolution on the meso level as the political, educational, industrial, societal, and environmental orchestration. On the micro level, makerspace populations provide services to maker individuals such as knowledge interaction, technology support, office location, political guidance, financial investment, business services, entrepreneurial advisors, and non-governmental community. In the knowledge ecosystem, makerspaces play the dual roles of resource orchestrator and platform supporter. As an incubated platform in the initial stage, makerspaces support the entrepreneurial services for startups through orchestrating multiple resources.

#### Makerspaces as the Role of Resource Orchestrators

Makerspaces define the resource orchestrators that invite the multiple related actors to share and create complementary resource for the value co-creation. A set of purposeful and deliberate interactive activities are integrated to build the knowledge ecosystem and then markets it. The role of resource orchestrator seems to be knowledge gatekeeper, intermediary, integrator, and builder to develop their knowledge base and stimulate its potential capabilities as a central agent. Makerspaces are vital to reinforce relationships such as political, educational, industrial, societal, and environmental orchestration for knowledge interaction through the knowledge community at the meso level. Clearly, the roles and functions of five agents could respectively impact the helical interaction across the levels, and in turn effect value co-creation in the knowledge ecosystem (Adner, 2017) (Table 6).

**Table 6:** The roles and functions of five agents in the helical interaction.

**Agents**	**Role**	**Function**	**Knowledge activities**
State/Government	Innovation supporter	Public funding, support program, stimulate consumption, and allocate resource	•Knowledge sharing, Knowledge discovery, Knowledge transfer, Knowledge creation, Knowledge acquisition, •Knowledge representation, •Knowledge dissemination, •Knowledge integration, •Knowledge orchestration
Academia/University	Proactive collaborator	Teaching, Knowledge development, Education, Offering advisors, Training	
Industry/Business	Reticular partnership	Partnerships, Investment, Donations, Job openings	
Civil Society	Shared participant	Participation, Collaboration, Employment, Empowerment, Memberships	
Natural environment	Resource trigger	Carrier of natural resource	Knowledge environment

##### Governments

*G*overnments and political sectors could initiate the essential resource of budgeting, the authority of resource configuration, the regulator of resource management, and resource preservation through public funding and support programs effectively (Lee et al., 2012). In China, government-driven resources are the main sort of coordinated effort by executives in public sectors. Government websites, network-driven advancement, and deliberate occasions are perceived as the acceptable incentive practices to help achieve ecological balance and monitor administrations. Online services, problem-solving initiatives, multi-sector collaboration, political administrations, social networks, crowd sourcing, EG (e-government), and government–university-business coordinated efforts are all progress forms of government support (Omar et al., 2017).

##### Universities

Universities play the role of proactive collaborator in knowledge ecosystem such as the educator of resource preservation, the exploration and R&D of unexploited resource and technology, and the research organization of resource utilization properly, rather than the traditionally educational organizations and scientific research institutions in the linear innovation model (Etzkowitz and Leydesdorff, 2000). There is increasingly more collaboration between strategic alliances and applied research among main agents for knowledge sharing and creation (Cai et al., 2019). A huge number of creative thinking emerges in universities but lack of practice and experimentation. Advisers and students can examine them with various sharing tools in makerspaces. Through related industrial market investigation, several innovative projects could be taken into practice, a startup project, and even a job. During R&D, makerspaces could consult professional advisers in the relevant domain from colleges (Hall et al., 2001).

##### Industries

Traditional industries appear to have a linear chain connection between upstream supply firms and downstream retail firms. In the knowledge ecosystem, companies cooperate as the user of resource, the practitioner of technology transfer and application. One or more hub firms act as leaders or facilitators that utilize their unique competitive advantages to construct a reticular ecosystem, and initiate collaborative innovation with other agents (Wang D. S., 2019). Regarding simulated systems, virtual prototyping evaluates environmental material from a sustainable development perspective, which conformed to the governmental request. More and more businesses embrace collaborative patterns as open platforms to cooperate with governments, universities, industries, and social or civil groups in addition to state-owned business and private business associated with empowerment, virtual multiple functions, and cross-product organization collaboration (Idelchik and Kogan, 2012; Wang R., 2019).

##### Civil Society

Civil society plays the role of shared participant to facilitate direct user and spreader of resource, the awareness and obligation of resource preservation, and cooperate to maintain ecological friendly environment. Citizens used by enterprises to capture consumer demands and adjust product attributes through client feedback and appraisal (Cohen et al., 2016). Social media is a common tool used to accelerate product consumption, promotion, and propaganda.

##### Natural Environment

Natural environment is paid more special attention in the Quintuple Helix model. The environment plays the carrier of potential of natural resources, and promotes the mutualistic symbiosis, coexistence, and co-evolution. The focus of Quintuple Helix theory is the new ecological helix of knowledge innovation. Makerspaces create knowledge production that is crucial for the utilization of the natural resource, the application of green and ecological technologies, environmental preservation, and co-survival of humans and nature.

#### Makerspaces as the Role of Platform Supporter

Makerspace is one of an incubated platform in the pre-startup phase, which usually provides the interpretative and packaged services of knowledge interaction, technology support, office location, political guidance, financial investment, business service, entrepreneurial advisor, and non-governmental community. As a platform supporter, makerspaces define and offer the basic knowledge architecture, which then become the platform or the foundation for other actors to build on through their own peculiar roles and functions in the knowledge ecosystem.

##### Governments

Based on experts interviews and surveys with makerspaces in China, this study finds that the government plays a role of policy window on the interactive activities through the support of makerspace platforms. This is crucial for makers and startups, which lack management experience, innovation capabilities, and market regulation (Omar et al., 2017). The public sector provides guidance by placing valuable information of public funding, programs supporting, stimulating consumption, and allocating resource through makerspace platforms (Ma et al., 2019). Governments offer special funding for the development of entrepreneurships and startups through incubated platforms for the whole ecosystem.

##### Universities

Since Premier Li Keqiang in China initiated the mass entrepreneurship and innovation program, there are increasing numbers of makerspaces emerging in the market. To cater to the developing market demand, some universities transform talent cultivation targets to entrepreneurs and startups that grow from academic achievements into commercialization, such as patent applications, out-licensing, and startups (Etzkowitz, 2001). Universities act in the role of teaching, training, project advising to transfer knowledge and technology to products (Ferasso et al., 2018; Teixeira et al., 2018), which creates collaborative relationships (Meyer, 2010; Jonsson et al., 2015). Various interactive mechanisms and patterns have been proposed in the university-industry collaboration with incremental changes through the support of incubation platforms.

##### Industries

While collaborative behavior can occur in large companies, makers or startups have some difficulty achieving external investment because of limited experience (Gan et al., 2019). However, startups are indispensable in the market because several innovative technologies and cutting-edge products are designed by startups (Lansiti and Levien, 2004; Omar et al., 2017). Large businesses often grow out of a startup. The funding from core firms can facilitate the innovative capabilities of startups by playing an active collaborative partnership by providing creative thinking in new products and specialized technological knowledge (Gassmann et al., 2010). Industries always play the role of collaborative partnership, project investment, and job openings for startup teams through makerspaces.

##### Civil Society

Civil society participates in knowledge ecosystem appears as crowdsourcing, crowdfunding, sharing comments, and memberships. Governments and businesses can use makerspaces to collect ideas or projects from the citizens (Berglund and Sandström, 2017). Crowdsourcing and crowdfunding are usually used to outsource programs to citizen through open ask. Citizens contribute to decision-making strategies of upstream companies and behavioral interests of downstream consumers based on sufficient capital (Wilson et al., 2018). Services and products can cater to market demands earlier and improve the response rate for consumers' demands in the sharing economy (Kapoor and Lee, 2013). Thus, crowdsourcing and crowdfunding speed up new product development flexibly and dynamically. Civil society includes not only passive consumers, but also participants in product research and development through makerspace platform (Prahalad and Ramaswamy, 2000; Chesbrough and Appleyard, 2007).

##### Natural Environment

The Quintuple Helix model demonstrates the relationship between eco-innovation, eco-entrepreneurship and sustainable development in a supportive environment for our future (Wang et al., 2018). Concerned with the effect on the natural environment, makers explore friendly environmental projects for promoting the potential of natural resource and damage reduction (Klemichen et al., 2022). Due to high-tech energy-saving development, startups cooperate with eco-friendly industries, which could be supported by governments. In the implementation of natural resources in makerspaces, the most important agents are still the universities, local industries, and government bodies. The sustainable value co-creation with economic and socio-ecological potential could be triggered by the natural environment.

**Figure 8:**
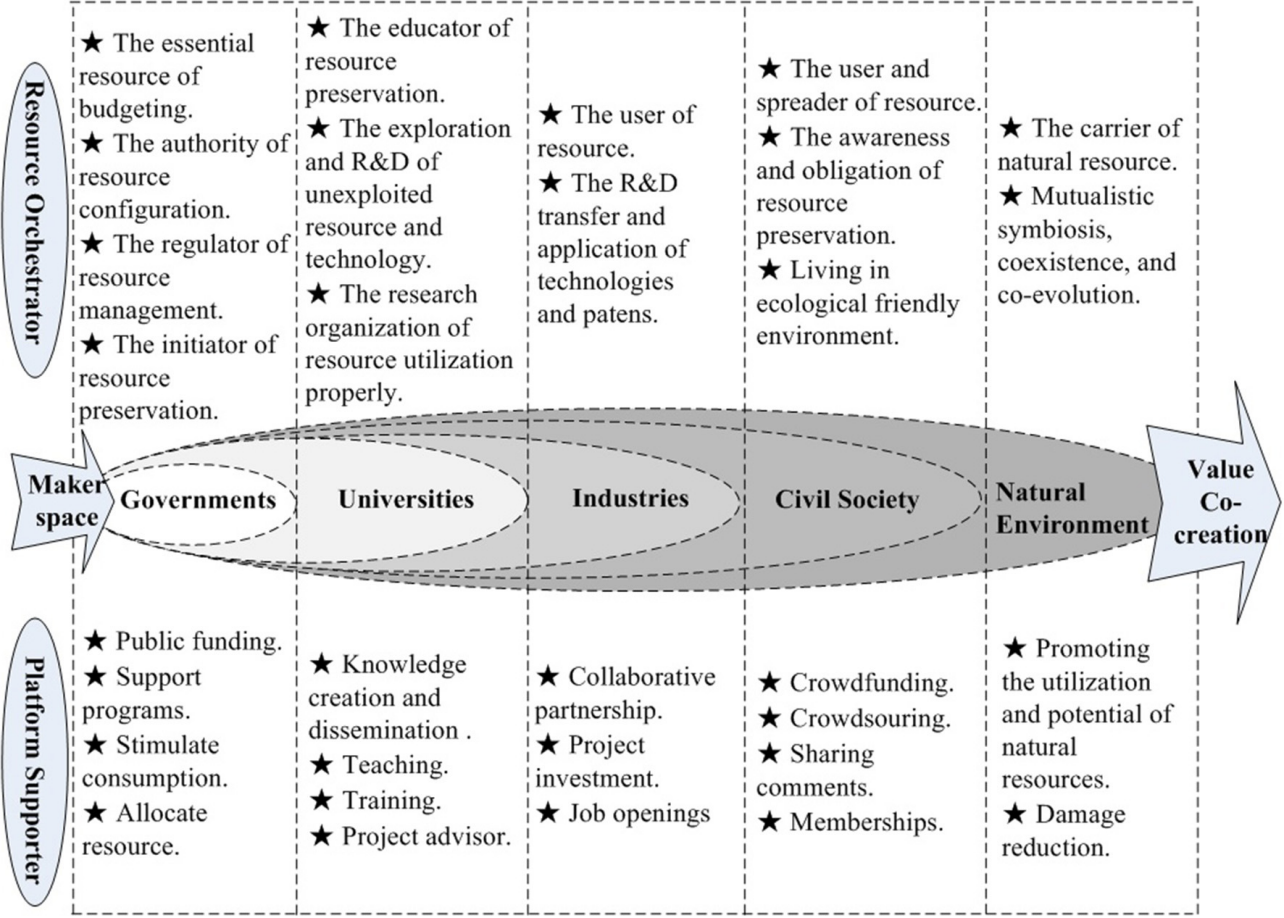
Makerspaces as resource orchestrator and platform supporter.

The roles of makerspaces and the functions of governments, universities, businesses, civil society, and natural environment for joint goal of value co-creation are summarized in Figure 8.

### Multi-Stage Orchestrating Process

Multi-stage knowledge orchestration aims to value co-creation interactively and gradually in the processes of knowledge orchestration among independent agents (Huang et al., 2020). This study explores the evolutionary stages of orchestrating processes based on the helix model, considering them as non-linear systems that rely on their agile, intertwined relationships (Baldwin and Von Hippel, 2011) and self-adaptability. The further proliferation requires multiple agents to provide the social, economic, institutional, political, and environmental resources for continuous linkages, and support the improvement of a collaborative, cohesive milieu. Therefore, the evolutionary processes of knowledge orchestration could be summarized as the four stages of knowledge exchange, knowledge coordination, knowledge cooperation, and knowledge orchestration (Al-kumaim et al., 2021). Particularly, interactive activities and different stages are distinguished by the extent of knowledge interaction and integration, such as exchange, coordination, cooperation, and orchestration, requiring agents to share joint knowledge-based activities for the purpose of value co-creation in a helix shape (Zhao, 2021). Compared to linear evolutionary stages of the past, the spiral model of orchestrating processes imply a dynamic and continuous changing balance among independent agents to meet fierce knowledge challenges and competition. Also, the spiral pattern is the focus of the Quintuple Helix model.

**Figure 9:**
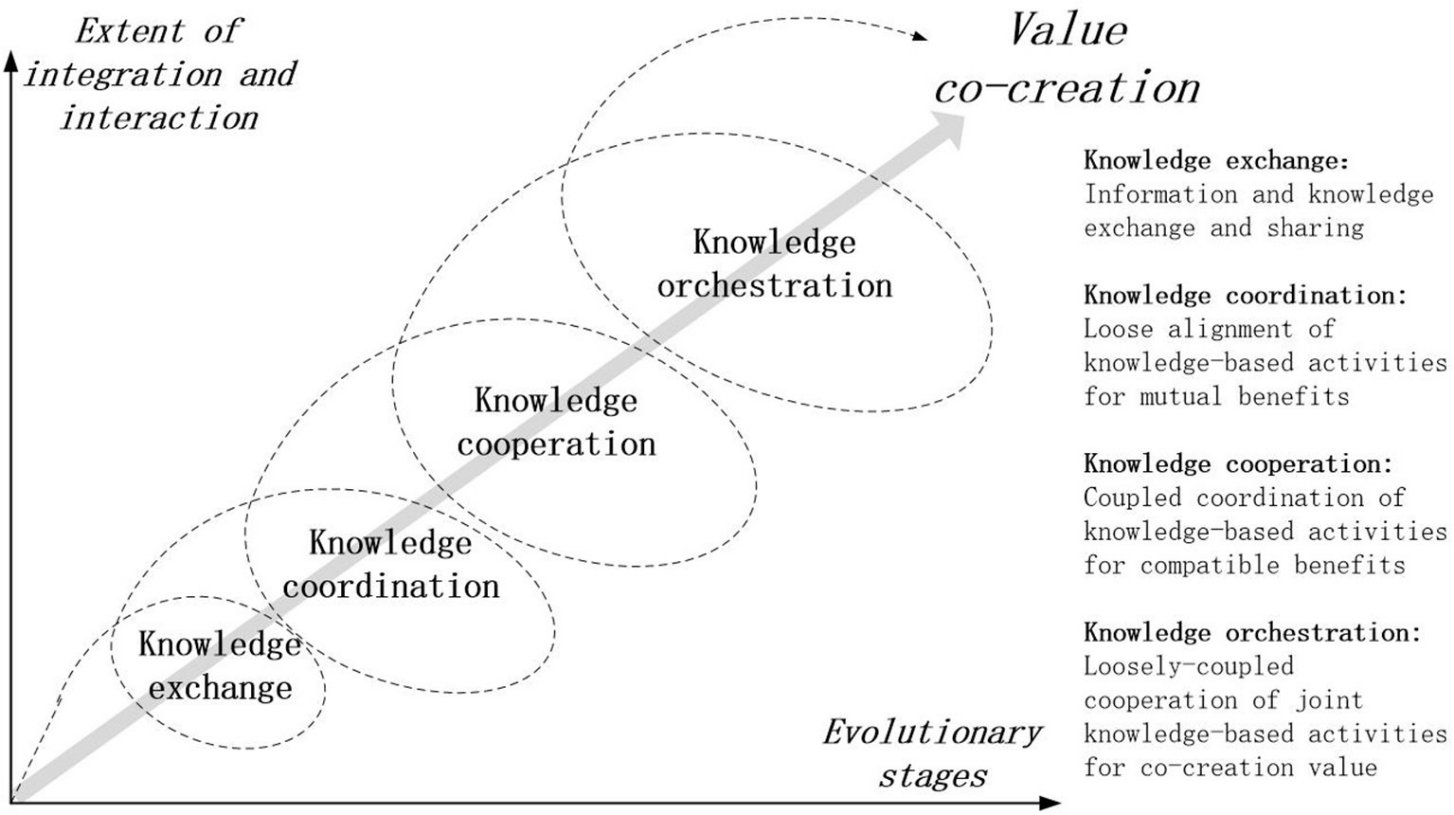
Multi-stage orchestrating process.

Figure 9 represents the evolutionary stages of orchestrating process. The first stage of knowledge exchange could be defined as information and knowledge exchange and sharing in the initial period. Such knowledge exchange could generate additional value for the whole knowledge ecosystem, and the activities enhance the appeal of new idea to makers and startups. Downstream, knowledge coordination is defined as loose coordinating stage of knowledge-based activities for mutual benefits. Then the stage of knowledge coordination gradually become coupled coordination for compatible benefits, which is called the third stage of knowledge cooperation. Finally, joint knowledge-based activities are loosely-coupled cooperation for value co-creation which is designed as the last stage of knowledge orchestration (Hong et al., 2020).

## Case Study

### Knowledge Exchange

For the initial period of knowledge exchange in China, makerspaces served makers as the platform of “interest clubs” and “innovation labs.” For examples, “New Workshop Makerspace” in Shanghai provided sharing experimental spaces and tools for makers to make their favorite technological products. Through knowledge exchanges freely, makers could combine creative ideas with active projects to the new product. “Firewood Makerspace” was a workshop of machine technology gathering in Shenzhen. The enthusiasts of open source hardware gather and communicate in makerspaces to exchange information and technology. “Beijing Makerspace” created a gathering place for new media artists and designers, and carried out various workshops and creative sharing sessions to provide a platform for makers in the fields of art design and new media technology. Makers with different interests and professional backgrounds put their creativity into practice through knowledge exchange and practice. “Onion Capsule Makerspace” in Hangzhou is the first domestic makerspace established by an art university. Makerspace provided knowledge exchange such as interactive art, new media technology, sound art, provided lectures, and training for makers. The makerspace becomes a communication platform focusing on art creation.

### Knowledge Coordination

For the further stage of loose knowledge coordination, makerspace began to develop rapidly based on knowledge coordination service for makers since 2015 in China. For examples, “Tsinghua X-lab Makerspace” provides various entrepreneurial training and cultivation services, and integrates interdisciplinary advisors for maker individuals who plan to establish a new project. Through the coordination of Tsinghua Entrepreneurs Association, and external cooperative enterprises, etc., makerspaces offer various innovation and entrepreneurship practice to help startups complete the business model in the pre-startup period. “Lenovo Star Makerspace” focuses on coordination with governments through public welfare entrepreneurship training and services to provide makers with financial investment such as “CEO Entrepreneurship Special Training Course.” Lenovo Star Makerspace builds a cooperation platform of knowledge exchange and cross-industry integration for entrepreneurs, and continues to share knowledge resources for entrepreneurs based on entrepreneurship alliance.

### Knowledge Cooperation

For the coupled stage of knowledge cooperation, makerspaces integrate the multiple resources tightly and closely for makers. “Tencent Makerspace” established in 2015 provided core knowledge resources with its own industrial platform and aggregated multiple knowledge resources in society to create a knowledge ecosystem. In recent years, the makerspace has pushed the sustainable development of ecological resources, and proposed the life cycle of start-ups. Tencent Makerspace integrated the internal and external complementary resources to enhance knowledge cooperation including community dissemination, industrial salon, Tencent Double Hundred Plan and some other venture capital. Through the coupled alliance of public sectors, incubation operators, venture capital, community participants, Tencent Makerspace constructs a multiple agent knowledge ecosystem platform to integrate the internal and external entrepreneurial resources. Makers could achieve the knowledge of emerging industries and cutting-edge technologies to cooperate with other actors and create joint value.

### Knowledge Orchestration

For the last stage of knowledge orchestration, let's take “Haichuanghui Makerspace” established by Haier consortium in 2014 as an example. The makerspace is an incubation platform for innovative and entrepreneurial projects served for Haier's industrial chain, and gradually developes to an ecological system of a crowd-creation sharing platform. Makerspace integrated the technological resources of U+ Smart Life, Zhongchuanghui, and Haidayuan to form a whole process of entrepreneurial knowledge ecosystem that takes connection and development strategies allying with some partner firms and research universities. The platform focuses on solving data mining, system simulations and modeling, and constructed virtual prototyping design in order to improve R&D capability (Wang D. S., 2019). Regarding simulated systems, virtual prototyping evaluates environmental material from a sustainable development perspective, which conformed to the governmental request.

## Discussion and Conclusion

This paper constructs the conceptual propositions of the multi-agent and multi-level knowledge ecosystem based on Quintuple Helix theory. The finding largely supports and elaborates on three main attributes of the ecosystem model, as follows: multi-level ecological balance, multi-agent helical interaction, and multi-stage orchestrating process. In order to sustain the ecological balance and interaction in the knowledge ecosystem, makerspaces play the roles of resource orchestrator and platform supporter for maker individuals and startups with other different agents. The Quintuple Helix model could be more appropriate than the Triple Helix model and the Quadruple Helix model. With the additional fourth helix of civil society and fifth helix of the natural environment, knowledge is more sensitive to “social ecology,” which comes together in the context of public citizens and the natural environment. Civil society involved in the knowledge ecosystem appears like crowdsourcing, crowdfunding and so on. The participation of civil society could help accelerate the degree of consumer satisfaction. The Quintuple Helix, furthermore, demonstrates what value co-creation might mean and imply eco-innovation and eco-entrepreneurship for our future, because the natural environment gets more attention to promote the utilization and potential of resources. In regard to the properties of makerspaces, four stages of knowledge exchange, knowledge coordination, knowledge cooperation, and knowledge orchestration are designed as the helix shape. We adopts several cases to verify the evolutionary stages of knowledge ecosystem such as *New Workshop Makerspace, Firewood Makerspace, Beijing Makerspace, Onion Capsule Makerspace, Tsinghua X-lab Makerspace, Lenovo Star Makerspace, Tencent Makerspace*, and *Haichuanghui Makerspace*.

Based on the Quintuple Helix model, this paper explains the model from macro, meso, and micro perspectives. At the same time, multi-agent orchestration, cross-level interaction, and evolutionary stages of knowledge orchestration are discussed in details. Makerspaces provides the direct practical basis and context for the construction of the multi-level and multi-agent ecosystem model. First of all, from the perspective of the ontology of ecological theory, makerspaces break the biological gap and form a multi-level framework which plays the role of knowledge element at the macro level, knowledge community at the meso level, and knowledge population at the micro level. Secondly, in terms of knowledge management theory, the multiple stages of knowledge exchange, coordination, cooperation, and orchestration fully demonstrates the evolutionary cycle of free exchange, loose coordination, coupled cooperation, and loose-coupled orchestration. Finally, from the Quintuple Helix perspective, the symbiosis, co-existence, interaction, and orchestration of multiple agents (governments, universities, businesses, media-based and culture-based civil society, and the natural environment) in the knowledge ecosystem should be analyzed for value co-creation at all levels of the knowledge ecosystem. Therefore, makerspaces require for the continuous attention of governments, scholars, business, citizens to make China's innovation and entrepreneurial strategy into an important engine to promote economic development and technological progress.

## Implication and Further Research

Our work has some implications for policymakers, managers, researchers. Makerspaces have strong linkages to governments, universities, businesses, citizens, and the natural environment. The activities of knowledge exchange, cooperation, collaboration, and orchestration are reinforced in the knowledge networks embedded by makerspaces. The implications from three aspects are consistent with the three levels of our conceptual framework. At the macro-level, makerspaces are elements of the knowledge ecosystem as a whole. The long-term run of makerspaces could structure the relationship of symbiosis, commensalism, intergrowth, and orchestration. The not-for-profit business model is ubiquitous and communal to the sustainability of the sharing economy. At the meso-level, makerspaces play the role of community, embracing diverse populations of makers, startups, and entrepreneurs. Makerspaces need a systematic approach based on the support by multiple agents to provide disparate services to new projects. At the micro-level, makerspaces provide the economic and social environment suitable for interactive knowledge-based activities. Makerspaces should construct more multiple and mature platforms for knowledge exchange and flow among the maker individuals, incubated startups, and the maker culture.

For further research, the case study should be extended to other countries, which may be more representative of all types of nations. Further research could compare our findings and practices with different contexts. Furthermore, some empirical data could be collected to verify our conceptual model.

## Data Availability Statement

The original contributions presented in the study are included in the article/supplementary material, further inquiries can be directed to the corresponding author.

## Author Contributions

All authors listed have made a substantial, direct, and intellectual contribution to the work and approved it for publication.

## Funding

This work was supported by the National Social Science Foundation of China (19BGL031).

## Conflict of Interest

The authors declare that the research was conducted in the absence of any commercial or financial relationships that could be construed as a potential conflict of interest.

## Publisher's Note

All claims expressed in this article are solely those of the authors and do not necessarily represent those of their affiliated organizations, or those of the publisher, the editors and the reviewers. Any product that may be evaluated in this article, or claim that may be made by its manufacturer, is not guaranteed or endorsed by the publisher.
